# Predicting the Fibroid-Migratory Impact of UAE: Role of Pre-embolization MRI Characteristics

**DOI:** 10.1007/s00270-019-02348-w

**Published:** 2019-10-24

**Authors:** Leto Mailli, Eric Y. Auyoung, Salvatore A. Angileri, Seyed Ameli-Renani, Lakshmi Ratnam, Raj Das, Joo-Young Chun, Sourav Das, Isaac Manyonda, Anna-Maria Belli

**Affiliations:** 1 Diagnostic and Interventional Radiology Department, St George’s University Hospital and NHS Foundation Trust, London, UK; 2 Diagnostic and Interventional Radiology Department, ASST Santi Paolo e Carlo, San Paolo Hospital, University of Milan, Milan, Italy; 3 Department of Obstetrics and Gynaecology, St George’s University Hospital and NHS Foundation Trust, London, UK

**Keywords:** Fibroids, Uterine fibroid embolisation, Uterine artery embolisation, Gynaecologic interventions, Fibroid MRI

## Abstract

**Aim:**

To investigate potential factors on MR imaging that could be used to predict migration of uterine fibroids post-UAE.

**Methods and Materials:**

We retrospectively reviewed patients referred for UAE having pre-procedural and 6 months post-procedural MRI, at a tertiary centre, over a 1-year period. Pre- and post-UAE images were reviewed in 64 women by two radiologists to identify the sub-type, dimensions, and infarction rate of each dominant fibroid. The shortest distance between the fibroid and the endometrial wall was measured to determine intramural fibroid movement. Paired sample *T* tests and two-sample *T* tests were used to compare between pre- and post-embolization variations and between migrated and non-migrated intramural fibroids, respectively. After preliminary results suggested potential predictors of intramural fibroids migration, we tested our findings against the non-dominant intramural fibroids in the same patients.

**Results:**

Review of images revealed 35 dominant intramural fibroids, of which eight migrated to become submucosal fibroids, while five were either partially or completely expelled. These 13 migrated fibroids had a shorter pre-procedural minimum endometrial distance (range 1–2.4 mm) and greater maximum fibroid diameter (range 5.1–18.1 cm), when compared to non-migrating fibroids. On image reassessment, the migrated non-dominant intramural fibroids had a minimum endometrial distance and maximum fibroid diameter within the same range.

**Conclusion:**

Intramural fibroids with a minimum endometrial distance less than 2.4 mm and a maximum fibroid diameter greater than 5.1 cm have a high likelihood of migrating towards the endometrial cavity after UAE.

## Introduction

Uterine artery embolization (UAE) is now established as a highly effective treatment for symptomatic fibroids [1–6], the commonest benign tumour in women of reproductive age [7, 8]. The available evidence on UAE confirms that the procedure is efficacious for symptom relief in a substantial proportion of patients [9], thus is now considered as an alternative to hysterectomy and myomectomy. Imaging protocols for UAE are well developed: there is a pre-procedural assessment by magnetic resonance imaging (MRI) followed by a post-procedural MRI 3–6 months later [10]. Various studies [10–14] have revealed that pre-procedural MRI improves patient selection and reduces treatment failure or complications, while post-procedural MRI allows for the assessment of treatment response or failure, in conjunction with symptomatic improvement.

Over a decade of performing UAE, we have observed that while the majority of fibroids will have undergone infarction on the MRI post-embolization, there are instances of fibroids showing a change in their location towards the uterine cavity, a phenomenon we have termed “fibroid migration”. Verma et al. [15, 16] have reported that in 1–5% of cases, fibroids do change locations: submucosal fibroids can become intracavitary and subserosal fibroids can develop an intramural or submucosal component—as in our own observations, the change in location appears to be predominantly towards the uterine cavity. This observation warrants careful study and understanding because it has potential clinical implications. It is likely to be the underlying mechanism behind the complication of fibroid extrusion and chronic vaginal discharge some women experience post-UAE. Perhaps even more importantly, fibroids changing location could impact negatively on reproduction. A fibroid migrating from an intramural position to an intracavitary position could represent one of the mechanisms of endometrial damage observed post-UAE [17], and intramural fibroids becoming submucosal could interfere with implantation. Thus an understanding of the factors associated with changes in fibroid location following UAE could enhance the quality of patient counselling and management. Those women wishing to conceive but where migration is likely could be advised surgical intervention, if it is proven that surgery has fertility benefits in such cases. Even if symptom relief is the predominant end-point for the planned treatment, they should be warned of the increased risk of fibroid expulsion per vagina [18].

Although studies have noted this change in location in intramural fibroids following UAE [18], our review of the literature failed to find published data evaluating characteristics that could predict these alterations. In this retrospective study, our aim is to assess imaging findings on pre-procedural MRI imaging in order to identify parameters that can be used to predict post-UAE migration of intramural fibroids.

## Materials and Methods

### Patients

This was a retrospective study of 73 consecutive patients with symptomatic fibroids referred for UAE at St George’s University Hospital NHS Foundation Trust between 1st June 2014 and 30th May 2015. The patients were identified through the Patient Archiving and Communication System (PACS) and had a mean age 45 years (range 30–60 years). Patients were required to have undergone a pre-procedural MRI followed by UAE and then a post-procedural MRI 6 months after embolization. Consequently, nine patients were excluded from the study because either the pre- or post-procedural MRI was not available for viewing, leaving a total study group of 64 women.

### MRI Technique

Pelvic MRI was performed with a GE Signa Excite (GE Medical Systems, USA) 1.5 T MRI scanner. Pre- and post-procedural MRIs were performed according to identical protocols.

Sequences acquired were as follows: sagittal T2 fast spin echo (FSE); axial T1 FSE; axial T2 FSE; and axial T1 fat-saturated postcontrast. Slice thickness of 5–6 mm was used with variable fields of view.

Contrast-enhanced sequences were acquired after intravenous injection of 20 mL gadolinium–DOTA (gadoteric acid; Dotarem, Guerbet, Obex). MRA was performed selecting the region of interest at the abdominal aorta and triggering image acquisition at approximately 25 s. Maximum-intensity projections of the abdomen and pelvis to the level of the renal arteries were reconstructed.

### Embolization Technique

The embolization procedure was performed by certified interventional radiologists in our institution via a bilateral transfemoral approach under local anaesthesia with selective catheterization of uterine arteries with 4F RIM catheters (Rösch Inferior Mesenteric Torcon Beacon Tip catheter, Cook Medical, USA). Two types of embolic agents were used: non-spherical PVA (nsPVA) (*n* = 16) 350–500 μm (Contour PVA, Boston Scientific, USA) and microspheres (*n* = 48) 700–900 μm (Embosphere, Biosphere Medical, Roissy, France). In both groups, if greater than four vials were required, the size of particles used was increased.

Embolization was performed in a standardized fashion: the RIM catheters were advanced to the contralateral internal iliac and uterine artery, sequentially over the aortic bifurcation and into the transverse segments of the uterine arteries, or as far as required to ensure a stable position. Microcatheters (Progreat 2.7F microcatheter, Terumo Medical Corporation, Europe) were used if the uterine arteries were small or the anatomy was unfavourable. Two operators carry out a bilateral hand-injected angiogram to confirm adequate position after which bilateral simultaneous injection of nsPVA or Microspheres was performed to achieve embolization with angiographic stasis of contrast within the transverse segments of the uterine arteries for approximately 10 cardiac beats. It was routine to cease embolization for 5 min, then subsequently reinject contrast bilaterally to confirm stasis of contrast in the transverse segments. If ovarian arterial collaterals were identified, catheter flush aortograms were performed after bilateral uterine artery embolization. Selective ovarian embolization was performed if the patient had consented prior to the treatment.

### Image Review

All MRI images were independently reviewed by two experienced radiologists with subsequent consensus review. On both pre-procedural and post-procedural images, the three-dimensional maximal measurements of the uterus and dominant fibroids were obtained using axial and sagittal T2-weighted MR images. In patients with multiple fibroids, the dominant fibroid was defined as the fibroid with the largest single dimension. Assuming an ellipsoid shape for both the uterus and the dominant fibroid, the volume was calculated in cubic centimetres using a prolate ellipsoid volume formula (0.523 × length × width × depth). The relative difference in volume was calculated and expressed as a percentage. In all post-procedural images, the percentage of fibroid infarction (non-enhancing fibroid tissue following contrast injection) was noted and categorized as 100%, 90–99%, 50–89%, or < 50%.

All dominant fibroids were categorized as being submucosal, intramural, subserosal, or endocavitary. A fibroid was defined as: submucosal, if any portion of the fibroid contacted the endometrium; intramural, if the fibroid was completely surrounded by myometrium; subserosal, if the fibroid partially or completely lacked a surrounding myometrium and abutted, or deformed, the serosal layer; and endocavitary, if greater than 50% of the surface area of the fibroid was surrounded by endometrium.

Additional measurements were obtained pre- and post-procedurally in the same plane on MRI for the dominant intramural fibroids by recording the shortest distance between the fibroid and the endometrial wall and between the fibroid and the serosal wall.

After obtaining preliminary results suggesting potential parameters to predict migration of intramural fibroids, we tested our findings against the non-dominant intramural fibroids in all our cases. We reviewed all images again and measured the non-dominant intramural fibroids as detailed above. We then determined if these fibroids had migrated and assessed for characteristic predictors.

### Statistical Analyses

Paired sample *T* tests were performed to assess pre- and post-embolization variations in the dominant intramural fibroid’s volume and maximal diameter. A two-sample *T* test was used to analyse parameters between migrated and non-migrated dominant intramural fibroids. Statistical significance was set to a *p* value of 0.05. Statistical power was not tested.

## Results

### Image Review Observations

Of the 64 dominant fibroids on pre-procedural MRI, 35 (55%) were intramural, 8 (13%) were submucosal, 20 (31%) were subserosal, and 1 (0.01%) was intracavitary. The mean maximal fibroid diameter of all dominant fibroids was reduced by 22.4% (range 0–100%; SD = 24.9; two-tailed *p* < 0.005; *t* = 5.4). The overall mean reduction in uterine volume was 40.1% (range 1.1–94.9%; SD = 20.8; two-tailed *p* < 0.005; *t* = 7.5) and the mean reduction in fibroid volume was 40% (range 0.4–100%; SD = 29.3; two-tailed *p* < 0.005; *t* = 3.4). Table 1 details the above parameters within each classification. Twenty-two (34%) patients had adenomyosis confirmed on pre-procedural MRI, but on post-procedural MRI, 15 (23%) patients were identified with adenomyosis. Complete infarction of dominant fibroids was seen in 40 (63%) of dominant fibroids, while seven (11%) were classed as 90–99% infarction, six (9%) as 50–89% and 11 (17%) as less than 50% (Table 2).

**Table 1:** Mean difference in maximal dimensions, uterine volume, and fibroid volume of dominant fibroids within classifications expressed as percentages

Dominant fibroid classification	No. (%)	Mean maximal fibroid diameter difference (%)	Mean uterine volume difference (%)	Mean fibroid volume difference (%)
Intramural	35 (55%)	23.4 (0–100)	41.8 (1.7–94.9)	39.6 (0.4–100)
Submucosal	8 (13%)	22.0 (1.3–47.1)	46.5 (24–89)	37.7 (3–83.1)
Subserosal	20 (31%)	21.3 (0–100)	35.1 (1.1–87.3)	42 (3.2–100)
Endocavitary	1 (1%)	14.3	27.1	35.7

**Table 2:** Infarction rates of dominant fibroids

Dominant fibroid classification	No. (%)	100% Infarction	90–99% Infarction	50–89% Infarction	< 50% infarction
Intramural	35 (55%)	26 (74.3%)	3 (8.6%)	2 (5.7%)	4 (11.4%)
Submucosal	8 (13%)	5 (62.5%)	1 (12.5%)	1 (12.5%)	1 (12.5%)
Subserosal	20 (31%)	9 (45%)	3 (15%)	3 (15%)	5 (25%)
Endocavitary	1 (1%)	0	0	0	1 (100%)

Fibroid migration was noted in 13 of the 35 dominant intramural fibroids (37%) with end-points as follows: eight became submucosal, two were partially expelled, and three were potentially completely expelled. The last three fibroids were assumed expelled as they were no longer visible at post-procedural MRI, and on review of clinical notes, vaginal discharge was described by the respective patients. Table 3 shows that in the 13 migrated dominant intramural fibroids, the mean pre-procedural minimal distance from fibroid to the endometrium was less than that seen with the non-migrating dominant intramural fibroids (two-tailed *p* = 0.01, *t* = 2.7). Furthermore, the mean pre-procedural maximum fibroid diameter of migrated dominant intramural fibroids was greater, with statistical significance, than those that did not migrate (two-tailed *p* = 0.02, *t* = 2.6). Although the mean fibroid volume of the migrated dominant intramural fibroids was also greater than the non-migrating fibroids, statistical significance was not reached (two-tailed *p* = 0.14, *t* = 1.6). All but one migrated dominant intramural fibroids were completely infarcted; the one exception being 90–99% infarcted.

**Table 3:** Comparison of pre-procedural dominant intramural fibroid measurements between migrating and non-migrating

Pre-procedural dominant intramural fibroid variables	Migrating dominant intramural fibroids (*n* = 13)	Non-migrating dominant intramural fibroids (*n* = 22)
Mean minimal endometrial distance (mm)	1.7 (1.0–2.4)*	10.2 (0.5–73)*
Mean maximal fibroid diameter (cm)	10.3 (5.1–18.1)*	7.2 (3.0–15.9)*
Mean fibroid volume (mL)	476.8 (57.6–2043.3)	189.7 (11.4–837.4)
Number with adenomyosis	5	6

*Statistical significance (two-tailed *p* < 0.05)

With respect to the eight dominant submucosal fibroids, we noted that one had migrated to become intracavitary and another to be partially expelled. The mean pre-procedural maximal fibroid diameter was greater in migrating dominant submucosal fibroids (mean = 7.2, range = 6.6–7.8) compared to non-migrating ones (mean = 5.5, range = 3.4–8.1), but this difference was not statistically significant. Both migrating dominant submucosal fibroids were completely infarcted on post-procedural MRI and the patient with the partially expelled fibroid had adenomyosis. Between the pre- and post-procedural MRI, one dominant subserosal fibroid was not evident on post-procedural MRI and assumed expelled. The one dominant intracavitary fibroid had poor infarction and did not appear to migrate.

Upon image reassessment of the non-dominant intramural fibroids, only four were found to have migrated. The mean minimal endometrial distance in these four cases was 2.05 mm (range 1.6–2.3), and the mean maximal fibroid diameter was 6.28 cm (range 5.1–6.8). All cases had complete infarction. Two of the fibroids migrated into a submucosal category, one was partially expelled, and one was completely expelled.

In summary, intramural fibroids with a minimum endometrial distance less than 2.4 mm and a maximum fibroid diameter greater than 5.1 cm have a high likelihood of migrating towards the endometrial cavity after UAE.

## Discussion

Patients undergoing UAE have comparable quality of life outcomes to hysterectomy and myomectomy with relatively minor complications [1–6, 19, 20]. Of the recognized complications, the passage of leiomyoma tissue, accompanied by symptoms of pain, bleeding or infection, as well as fibroid impaction are likely to be a direct result of fibroids migrating into the uterine cavity [21]. Fibroid migration could also be a mechanism for the endometrial injury reported by Mara et al. [17] as a consequence of UAE; this in turn may compromise fertility potential. Therefore an understanding of the underlying processes, mechanisms, and sequelae is important for the appropriate counselling of patients and their management.

**Fig. 1:**
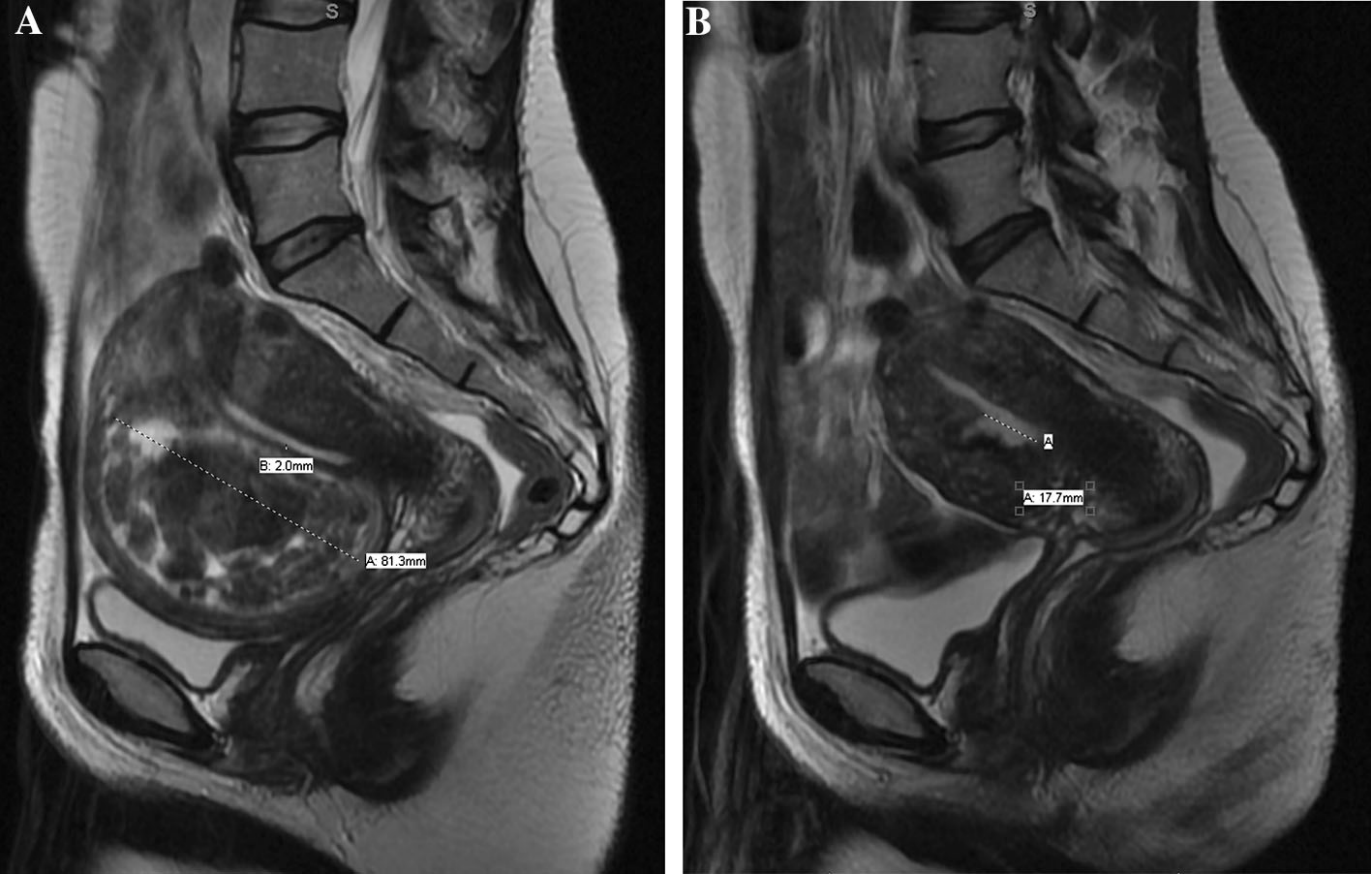
**A** Sagittal T2-weighted MR image shows an intramural fibroid measuring 81.3 mm in long axis with 2 mm shortest distance to the endometrium. **B** Repeat MRI 6 months following uterine artery embolization demonstrates a 17.7 mm discontinuity in the endometrial lining. This was interpreted as an expelled fibroid given the imaging and clinical correlation of tissue-like discharge described by the patient

Our results have demonstrated that 37% (*n* = 13) of dominant intramural fibroids underwent migration to become submucosal or were expelled. Our measurements showed that only these 13 migrated dominant intramural fibroids had a pre-procedural minimum distance from the endometrium of 1–2.4 mm, and of these, all had a pre-procedural maximum fibroid diameter greater than 5.1 cm (Fig. 1). Since this finding suggested that an intramural fibroid with a pre-procedural minimum endometrial distance of less than 2.4 mm and a maximum fibroid diameter greater than 5.1 cm will migrate to either become submucosal or be expelled, we decided to test this hypothesis on the non-dominant intramural fibroids in our unselected patient population. Coincidentally, all the non-dominant intramural fibroids that underwent migration also fell under these parameters. It has previously been postulated that fibroids with a portion of its circumference contacting the endometrium can be expelled [22]; however, our study shows that those within 1–2.4 mm distance to the endometrium also predisposes an intramural fibroid to migrate towards the uterine cavity. We do not know of the anatomical or physiological explanation for the fibroids migrating towards, rather than away from the uterine cavity. It may be that the reduction in the uterine volume pushes the large fibroid towards the endometrial wall; another possibility is that the ischaemic change caused by the embolization damages the adjacent endometrium resulting in expulsion of the fibroid through the damaged layer. However these explanations are hypothetical and not proven.

How do our findings compare to previous reports? While we found that 37% of dominant intramural fibroids underwent migration to become submucosal or expelled, Verma et al. [16] reported a much smaller percentage (4%) of dominant intramural fibroids changing to a submucosal location after UAE. It is not immediately apparent as to why there should be such a large difference between the two studies, but there were differences in study design. Our study sample included only the dominant fibroid in each patient, whereas Verma et al. [16] had evaluated between one and five fibroids per patient. The submucosal fibroid parameters of pre-procedural interface–dimension ratio of 0.55–0.83 and a maximum fibroid dimension of 3–17 cm were observed by Verma et al. [16] to be more likely to become intracavitary after UAE. We had one fibroid that met these measurements; however, it was not found to have migrated to an intracavitary position. Instead, the fibroid in our study that migrated to become intracavitary after UAE had a maximal fibroid diameter of 6.6 cm and an interface–dimension ratio of 0.94. More samples and further studies can likely assess the reproducibility of the parameters suggested by Verma et al. [16].

We acknowledge the limitations in our study consequent upon the small sample size of 64 patients. However, our data can be considered as preliminary results revealing a correlation between dominant intramural fibroid migration, pre-procedural distance to endometrium, and pre-procedural maximum fibroid diameter. While measuring the minimum distance to endometrium may be difficult to identify in some cases, we believe that in most cases, it can be easily assessed with routine measurements of dominant uterine fibroids. In our study, post-procedural MR images were obtained after 6 months. Future studies may assess whether a scan more than 6 months after UAE may reveal the extent of migration in the dominant intramural fibroids that became submucosal.

## Conclusion

In this pilot study of dominant fibroid migration, we found that dominant intramural fibroids have a high likelihood of migrating towards the endometrial cavity if the pre-procedural MRI minimum distance to the endometrium is less than 2.4 mm and the maximum fibroid diameter is greater than 5.1 cm. Additional research in this area is required, not least to test the reproducibility of our findings, and further define other parameters that could enhance the predictive potential of pre-UAE MRI fibroid characteristics.
